# Patient perspectives about treatment preferences for obesity with complications

**DOI:** 10.1002/osp4.720

**Published:** 2023-10-29

**Authors:** Hilary C. Craig, Dalal Alsaeed, Suzanne Norris, John Holian, Cormac Kennedy, Alix Feldman, Carel Le Roux

**Affiliations:** ^1^ Diabetes Complications Research Centre UCD Conway Institute of Biomedical and Biomolecular Research School of Medicine University College Dublin Dublin Ireland; ^2^ Dasman Diabetes Institute Dasman Kuwait; ^3^ Hepatology Department St James's Hospital Dublin Ireland; ^4^ Nephrology Department St Vincent's University Hospital Dublin Ireland; ^5^ Hypertension Clinic St James's Hospital Dublin Ireland; ^6^ Global Medical Affairs Bagsvaerd Denmark

**Keywords:** chronic disease management, integrated care, lifestyle therapies, obesity, pharmacotherapies, self‐care, stigma

## Abstract

**Objective:**

Obesity and many of its comorbidities can be improved by nutritional therapy, lifestyle modification, pharmacotherapy, and surgical intervention. Relatively little is known about patients' preferences for the range of obesity treatments. The present study was undertaken to identify factors that may influence these preferences. By evaluating patient‐preferred treatment options and factors influencing patients, treatment adherence and efficacy may be improved. Our objective was to identify factors that influence patient preferences and subsequent choice of obesity treatment among those seeking treatment for obesity‐related complications.

**Methods:**

Participatory action research, using purposeful sampling, was used to recruit 33 patients with obesity complications. Recruitment took place in specialist clinics for non‐alcoholic fatty liver disease, diabetes, hypertension, and chronic kidney disease. Sixteen males and 17 females aged 18–70 years with a BMI>35 kg/m^2^ were recruited. Prior to the interview, participants watched a 60‐min video explaining nutritional therapies, pharmacotherapies, and surgical therapies in equipoise. Data were collected in one‐to‐one semi‐structured interviews using zoom or the telephone; reflective thematic analysis was used.

**Results:**

Four themes emerged: 1) structural factors, 2) autonomy, 3) interaction with formal care, and 4) the emotional and physical consequences of obesity. 39% of participants preferred nutritional therapy with support from medical professionals. 27% chose bariatric surgery. 24% chose pharmacotherapy alone, while 6% chose pharmacotherapy combined with nutritional therapy, 3% of participants wanted no intervention.

**Conclusion:**

The challenges can be addressed by increasing support for healthcare professionals toward enhancing both their knowledge and the health literacy of patients. Future research should focus on improving access to treatment pathways for patients as well as developing health literacy programs and educational programs for healthcare professionals.

## Graphical abstract

By evaluating patient‐preferred treatment options and factors influencing them, treatment adherence and efficacy can be improved. This study found that the factors which influence decisions around treatment options for obesity complications include structural factors of healthcare setting, autonomy, interaction with formal care, and emotional and physical consequences of obesity. These challenges can be addressed by increasing support for healthcare professionals toward enhancing both their knowledge and the health literacy of patients. Future research on health policy should focus on improving access to treatment pathways for patients as well as developing health literacy programs and educational programs for healthcare professionals.

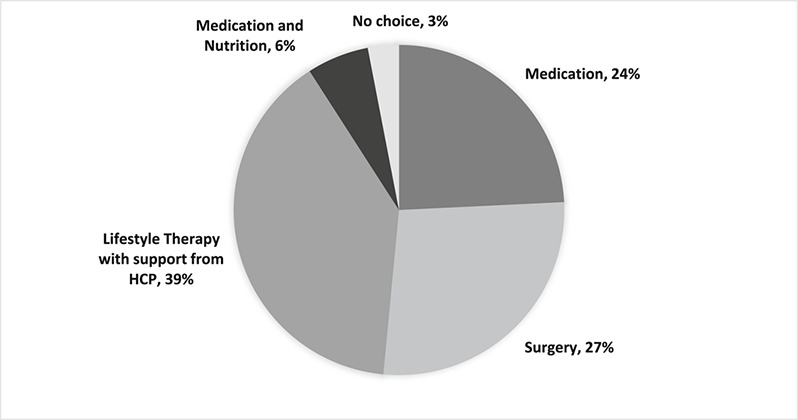

## 1 INTRODUCTION

The components of evidence‐based medicine include the best available evidence and clinical expertise, as well as taking a patient's values and circumstances into consideration. These components are essential to provide a patient focused, optimal treatment plan. Patient preference is one of the foundations of patient‐centered care and is informed by the patient's beliefs, values, expectations and goals for their health.^1^, ^2^ Understanding decision making and the importance of patient's values and goals has become an essential part of the provision of quality care.^3^ Involving both healthcare providers and patients in establishing a patient specific obesity treatment plan has the potential to lead to adherence to treatment and better health outcomes.

Patient preferences include the process that patients use in deciding the benefits, harms, and management of different treatment options.^1^ They are complex, as people can make decisions from different viewpoints that may encompass emotional and social influences.^1^ Understanding patient preferences for patients with obesity complications may impact treatment planning to make interventions more effective.^4^ Moreover, this may improve patient engagement in treatment adherences and can reduce costs as patients become more compliant with treatment.^5^ In clinical practice, significant challenges remain as regards effective communication, knowledge gaps, and patients' experiences of not being heard.^4^ It is difficult for patients to choose treatment options if they do not have the knowledge to understand what is available or if they have insufficient support from their health provider to manage their obesity and obesity complications. The barriers to the treatment of obesity and obesity complications are thought to include a lack of knowledge, a lack of access to services, as well as the prohibitive cost of treatment.^5^, ^6^ Understanding what motivates patients to engage with treatment options may provide the key to unlocking these barriers to effective treatment of obesity and obesity related complications.^6^

There is a paucity of research regarding patient preferences for the treatment of obesity and its complications. Previous research on patient preferences among those with obesity‐related comorbidities has identified themes related to lack of information and support and a need for more tailored treatment programmes.^7^ As obesity is a complex disease, there is a growing awareness of the need to provide treatment that reaches beyond lifestyle intervention. The objective of this study was to evaluate patient preferences regarding treatment options and factors which influence treatment choice.

## 2 METHODS

To achieve an in‐depth understanding of patient perspectives, participatory action research (PAR) was used to facilitate the co‐construction of knowledge through collective reflection and investigation between researchers and participants.^8^ Participatory action research involves the sharing of findings with participants and collectively promoting change.^9^ We aimed to establish a greater understanding and knowledge of obesity and treatment options among patients, capturing the participant voices and advocating for positive change. The initial data collection was analyzed through reflective thematic analysis over a 6‐month period. The interview questions were discussed with the research team and developed using previous research as well as experiences from the Stratification of Obesity Phenotypes to Optimize Future Obesity Therapy (SOPHIA) project. The interviews began with general questions followed by specific questions surrounding treatment preferences. This elucidated the motivations and factors influencing patient decisions. The interviews ranged between 30 and 45 min for an in‐depth exploration of participants' views.

The overall approach was to establish the participants understanding and knowledge of their condition and their choices, capturing the participant's voices as well as advocating for positive change. The participants received an informational video which explained all the treatment options in equipoise. Comprehensive lifestyle changes, including healthy eating and healthy exercise, were the cornerstone of all treatment options. In addition, patients were provided with information on specific treatments for the disease of obesity, including nutritional therapies, pharmacotherapies, and surgical therapies, by a specialist dietician, obesity medicine physician and bariatric surgeon. The initial data collection was analyzed through reflective thematic analysis. The data collected will also be part in the future of a triangulation strategy to validate each piece of data against others collected with different methodologies,^10^ where data collected separately will be given equal weight.^11^ Particular features of this design that are especially important are participation, collaboration and practical aspects and that the participant is treated in an equitable way. This study takes a rights‐based approach, which is the mechanism to put human rights at the forefront of any policy or process. A rights‐based approach to health policy advocates for more equity and more participation.^12^, ^13^

### 2.1 Recruitment

Purposeful sampling was used to recruit 33 patients with obesity complications. Creswell (2012) describes purposeful sampling as the researchers purposefully or intentionally selecting the individuals and sites as the best way to understand or learn about the phenomenon.^9^ The standard way in which to choose the participants and sites is where they are ‘information‐rich’.^9^ Recruitment took place in specialist clinics for non‐alcoholic fatty liver disease, diabetes, hypertension, and chronic kidney disease. None of the participants had been previously treated for obesity. Sixteen males and 17 females aged 18–70 years, all with a BMI >35 kg/m^2^ were recruited.

### 2.2 The interview

Due to COVID‐19 restrictions, data were collected in one‐to‐one semi‐structured interviews using zoom or over the telephone. Interviews were carried out by the first author, who built a relationship with this participant group through recruitment in the relevant specialist clinic. Prior to the interview, participants watched a 60‐min video featuring three medical experts in obesity treatment from University College Dublin explaining nutritional therapies, pharmacotherapies, and surgical therapies. The information was provided in equipoise, highlighting the advantages and disadvantages of each treatment (https://www.itsnotyourfault.ie/research).

### 2.3 Data analysis

The interviews were recorded and transcribed verbatim. A coding framework was developed by the first author based on previous research on the topic and the interview transcripts. Transcripts were anonymized and added to MAXQDA 2022 plus software to aid the coding of the data. Reflective thematic analysis was conducted by the first authors, and an inductive approach identified and reviewed the themes and sub‐themes within the study.^9^, ^14^ The data was interpreted through a social cultural lens to understand the factors influencing the participants' choice, including motivations and the impact of obesity complications. In addition, content analysis was used to ascertain the percentage of participants who stated their intention to choose between the available obesity treatments.^15^ Based on discussions with all authors, codes and themes were refined and agreed upon using an iterative approach to foster reflexivity and dialog, and consensus was achieved. Ethical approval was obtained from the Human Research Ethics Committee‐ Sciences (HREC), University College Dublin, Ireland 6 August 2021.

## 3 RESULTS

### 3.1 Treatment choice

**FIGURE 1:**
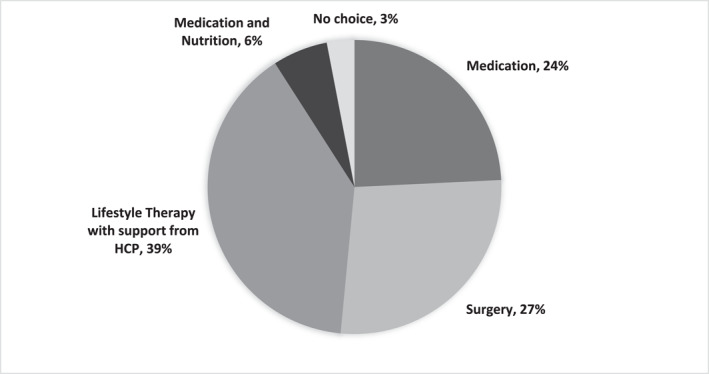
Participants' obesity treatment preferences.

Table 1 shows the identified themes and sub‐themes. Figure 1 shows participants obesity treatment preferences. Figure 1 shows 39% of participants preferred nutritional therapy with support from a medical professional. Although participants did not perceive dietitians as being particularly supportive, the participants believed that a medical professional, such as a nurse or doctor, would be able to help them with nutritional therapy. Among participants, 27% chose surgery, although access to this treatment was a challenge that presented as a theme. Pharmacotherapy alone was chosen by 24% of participants, while 6% chose pharmacotherapy combined with nutritional therapy. No intervention was desired by 3% of the participants.

**TABLE 1:** Identified themes and sub‐themes.

Themes	Sub‐themes
Structural factors influencing choice	Access
	Cost
Autonomy	Knowledge and information
	Not being heard
Interaction with formal care	Knowledge and information
	Support
	Consistency and integration of care
Emotional and physical consequences of obesity	Emotional impact
	Physical impact
	Support—Family and friends
	Side‐effects

### 3.2 Identified themes

Four themes emerged from the reflective thematic analysis to determine the preferences for obesity treatment and the factors that influence choice: 1) structural factors of healthcare setting, 2) autonomy, 3) interaction with formal care, and 4) emotional and physical consequences of obesity. These themes are shown in Table 2.

**TABLE 2:** Questions to participants.

1.	What did you think/expect when you were asked to take part in this study*? (Ice breaker is to establish rapport)*
2.	You have been diagnosed with NAFLD/T2D/CKD etc. Can you please tell me a bit about your experience of living with NAFLD/T2D/CKD? a. Ask follow‐up questions based on what they describe chronic conditions
3.	What health benefits do you think you may gain with weight loss?
4.	Did your doctors ever discuss management options to help you lose weight? a. If yes, what were those options? And what were your thoughts about them at the time? b. If not, why do you think that?
5.	Have you ever asked any of your doctors to help with weight loss? a. If yes, what happened? What led you to seek help? b. If not, why not? (Prompt about stigma, feelings of blame, access to care, low concerns)
6.	You have had a chance to watch the video that was shared which spoke about the different treatment options available. Which treatment would you prefer? c. Why did you choose that treatment? d. What are your expectations of the treatment you chose? e. Why did you not choose one of the other treatments?
7.	What would have to change for you to choose nutritional therapy/pharmacotherapy/surgical therapy? *(Specify the treatments they did not choose)*
8.	Is there anything else you feel we missed in our discussion that you would like to add?
	Thank you for your time
Generic prompts	

Could you explain further?	What do you think are the reasons for?
Can you give an example/s?	What other issues are important regarding this?
In what ways?	How often?
Why do you think?	I would now like to move on to a different topic

### 3.3 Theme 1: Structural factors of healthcare setting

This theme is related to the health systems and structured health processes that enable people to obtain treatment. Participant treatment choice had two sub‐themes: 1) access, 2) cost.

#### 3.3.1 Access

Many participants described having limited access to information about treatment, as well as how to access these treatment options. Before being presented treatment information in the context of this study, several participants indicated a lack of understanding regarding available options and were unsure about the available pathways for treatment access. Those who obtained treatment referrals often waited 4–8 years for treatment due to significant wait times in the public health system. Thus, the participants had very serious uncertainty regarding their treatment pathway. For lifestyle therapies, participants noted that, after the initial meeting with a dietitian, the participants often waited several years for a follow‐up or did not receive a follow‐up session.

‘The hospital did but it never came to anything…Three years later I’m still waiting, like the dietitian could help you with what to eat’ Patient with NAFLD

Concerns were raised in terms of access to anti‐obesity medication, with patients reporting easier access though diabetes care pathways but difficulties though non‐diabetes care pathways for example, NAFLD and/or CKD clinics.

‘I knew my GP wouldn’t give it to me or wouldn’t tick a box to say I was pre‐diabetic so that’s why I asked the hospital and part of me was hoping I’d have a slightly raised blood sugar level… So, like I begged them for it, and she gave me all the horror stories and that I have to keep going and you have to practice better health…then I paid €150 a month.’ Patient with NAFLD

#### 3.3.2 Cost

The cost of treatment concerned many participants, particularly the cost of medication, which many found prohibitive. Even when prescribed anti‐obesity medications, some individuals did not adhere to treatment as the participants could not afford it.

‘The tablets or the injection was okay, but I wouldn't be able to afford to pay for it… Because I didn't want to really go for the surgery, but because it was so much money, I said I'll just go for the surgery, and then hope that everything works out…Patient with CKD

The cost of surgery and after‐care was also a concern, which prevented many from considering this option. Follow‐up in primary care was also cited as costly and often occurring only at the instigation of the participant and not the clinician.

### 3.4 Theme 2: Autonomy

The participants' ability to exercise their right to choose their treatment preference had two sub‐themes 1) knowledge and information and 2) the perception of not being heard by healthcare professionals.

#### 3.4.1 Knowledge/information—Lack of awareness about treatment options

Many participants felt that their own knowledge about treatment options was limited and that there was not enough information available. After watching the informational video, some participants reported changing their treatment preference as they had more information and a better understanding of available options. For several participants, it was perceived that care decisions were made without their involvement or understanding.

‘It’s a big leap into the unknown. And obviously I’d like more knowledge and maybe more research into all of these things.’ Patient with NAFLD

#### 3.4.2 Not feeling heard or included in decision making

Many participants did not feel able to instigate a discussion about obesity treatment with their healthcare professional. Participants did not believe that they were being sufficiently listened to during these conversations. Some participants experienced this lack of effective communication as a personal failing. The participants described being dismissed with the suggestion that they should ‘try harder’, placing the responsibility on their shoulders. The participants internalized this into self‐blame and chose not to communicate any further with the healthcare professional.

’…you're looked at as if it's all completely your fault. I know some of it is, you know what I mean. It could be 70% your own fault. But when you're asking for help it's kind of like, “Just go away and do that, and then come back to me.” …When you're struggling to do it, you get frustrated with yourself, and you give up. And it gets hard.’ Patient with CKD

### 3.5 Theme 3: Interaction with formal care

Interaction with formal care, as a theme affecting treatment preference, was subdivided into 1) knowledge and information, 2) support, and 3) consistency and integration of care.

#### 3.5.1 Knowledge and information

Participants felt that their healthcare professional's views were an important influencing factor. However, the participants also believed that their healthcare professionals lacked information about treatment options for obesity.

‘I was just told, go to Weight Watchers, start losing weight. I was told to Google it, have a look online. It is very difficult to do that when you’ve got, when you don’t really understand what you are reading.’ Patient with T2D

#### 3.5.2 Lack of support

Participants reported the need for support for obesity treatments. For example, among those who saw a dietitian, many only had one session, where the participants were handed a food pyramid. The participants expressed a desire for consistent engagement with a healthcare professional–such as an advanced nurse practitioner or general practitioner–every few months to assist with treatment management. Participants expressed a desire for emotional and physical health monitoring alongside lifestyle interventions. Some participants had concerns that, while on medications, the participants were ignored with their disease management once they were returned to primary care. One participant was on medication without follow‐up for 2 years.

‘I'm just saying that it's the comfort and reassurance of being able to pick up the phone and say, listen, hi, something is not going well or I'm gaining weight for some reason or something's happening… Do you know that they could pick up the phone and not be judged either, but also, you know, I suppose that's all about the stigma of weight… I seem to not be losing weight. And rather than them feeling ashamed it'd be like, oh yeah, okay, come on in now, you know, come in on Wednesday… I definitely think even a, like I said, a nurse or someone else from the team or whatever.’ Patient with CKD

#### 3.5.3 Consistency and integration of care

Many participants raised their concerns about the lack of a multi‐disciplinary approach to management and care. Often, the participants visit multiple clinics for different complications and find a lack of coordination in their healthcare.

‘*I’m not entirely sure how the whole system works, because I’m seeing different people maybe you can explain it to me. Different people every time I go and visit for one reason or another, they all seem to refer to the written notes and they seem to be that there's an overlap, essentially, it seems the initial visits were very beneficial, and I learned a lot, but now it's essentially repetitive they go over the same ground’ Patient with NALFD*

### 3.6 Theme 4: Emotional and physical consequences of obesity

The theme of emotional and physical consequences consists of 4 sub‐themes: 1) emotional impact, 2) physical impact, 3) fear of side effects, and 4) support from family and friends.

#### 3.6.1 Emotional impact

The emotional impact of obesity and associated complications had a significant influence on participants' choice and behavior. Some participants reported that their personal weight challenges left them with feelings of depression, shame, worry, anxiety, and low self‐esteem. This manifested in self‐stigmatization. Participants became demoralized about their obesity and related complications, leading to a lack of hope and/or a sense of futility, which influenced engagement with their healthcare professionals.

‘Weight affects absolutely every part of life. It degrades people in their own mind. It puts you to the level zero, takes couple of years to gather energy to lift up from there, you feel good for a while, but then your brain brings you down again,’ Patient with NAFLD

Several participants described how their life was limited by the complications of obesity, and how they viewed the need of frequent healthcare visits as a constant reminder of their restrictions. Participants compared their own weight loss journey with others, which often had an emotional effect.

‘When you are 120kg you have access to everything… you could still say I don't care about myself anymore, because I am not living my life, I'm living a life of this misery you feel in your body and every time you do something about it, it's kind of somewhere at some level you fail and you go back to the same thing… You live something, some kind of battle, that you and your hypothalamus and everything else in your body is, you're trapped with it. So, the other thing you need something that allow you to get a grip and fight.’ Patient with NAFLD

#### 3.6.2 Physical impact

Participants reported that their mobility issues impacted their treatment choices. Exercising and taking part in daily activities–particularly with children–were challenges. This influenced the decision regarding treatment choices, depending on the severity of physical impact.

‘I know me weight has causing a lot of problems with my health as well, because I suffer with me back and knee and ankles and having awful trouble, so I need to lose weight’ Patient with NAFLD

#### 3.6.3 Fear of side effects

Participants' fear of medication side effects and their perceived severity influenced choice. However, the health benefits and health improvements in response to treatment were stated as motivations for trying pharmacotherapy. Fear of the treatment itself was also a factor. For surgery, participants had fears about the actual procedure and its after‐effects.

‘Like for me 100% no way I would go under the knife. And like the medication… I said to myself no way who wants to have to pay them for medication that reacts to the fats in your body giving you diarrhoea and all that no way, no way I said would I do that.’ Patient with NAFLD

#### 3.6.4 Support from family and friends

Support from family and friends was vital for treatment choices, as well as improving the quality of life and helping to manage obesity‐related complications. Some participants expressed how their family's lack of support had a severe effect, as participants had to deal with judgments surrounding their appearance. Participants reported feeling demoralized and acquiring a self‐belief that, no matter what treatment they chose, they were always going to be ‘big’. When participants were supported, they engaged better with healthcare professionals. However, without support, participants either retreated or were anxious about treatment.

‘I remember going in for Chemo and like one of the first things my mother asked them was…does she need to lose weight… before I go in for chemotherapy because that’s what I’ve lived with all my life.’ Patient with NAFLD

## 4 DISCUSSION

The objective of this study was to evaluate patient preferences regarding treatment options and factors influencing treatment choice. The lived experiences of patients with obesity‐related complications highlighted the paucity of obesity‐related knowledge, demoralizing or inconclusive interactions with healthcare professionals, and limited follow‐up care. This underlined the need for improved communication, supportive consultations with healthcare professionals, and longer‐term support and follow‐up care.

The lived experiences of these participants are aligned with the current understanding that obesity is a complex disease. Following their participation, patients gained a better understanding of the need for a multi‐disciplinary approach toward nutritional therapies, pharmacotherapies, and surgical therapies. Patient preferences indicated that nutritional therapies are still the most popular, while the cost, access, and side effects of pharmacotherapies and surgical therapies remained a barrier.

Patient‐centered care enhances a patient's autonomy and allows patients to participate in discussions and decision making based on their values and needs.^16^ Queally et al (2020) outlined the importance of patient preferences regarding treatment choices and decision making to develop services if compliance is to be maximized.^17^ In this study, some participants reported feeling dismissed, leading to ineffective and disengaged interactions with healthcare professionals. For patients with obesity, this is particularly important as participants often experience shame and stigmatization surrounding their disease, making patients reluctant to seek help.^18^, ^19^, ^20^ Previous qualitative studies on patient preferences have described how the discussion of how to manage weight in the patients' medical consultations was very infrequently conducted. Patients in these studies reported that they felt this was because they were not worth the medical time.^21^ In their systematic review of patient preferences: clinical encounters about obesity: Ananthakumar T. et al (2019) found that patients‐negative experiences in consultations related to ‘snag judgments and flippant advice’(21). Patients were concerned that doctors assumed that symptoms were because of weight and so may miss a serious illness without a proper examination.^21^ Sarwer et al (2021) described the option of shared decision making where all parties share information and work to come to a treatment decision. The share decision making may help patients understand the risks and benefits of treatment options and all information to make an informed decision as well as having the opportunity to express their preferences for treatment.^22^

Limited knowledge about treatment options and the perspective of not being heard influenced participants' choices and their engagement in the healthcare pathway. Healthcare professionals and patients reaching a consensus on optimal obesity treatment and follow‐up management may lead to better adherence and better health outcomes.^16^, ^23^ The lack of knowledge among healthcare professionals about obesity treatment options was a barrier to patients. Patient‐centered care focuses on the patient and their health needs, which translates into greater adherence to prescribed treatment, greater satisfaction and improved health outcomes.^24^ Several participants reported the need to have consistent support with their chronic disease management. Participants viewed their care as fragmented and believed that meeting every few months with a healthcare professional, such as a nurse or general practitioner, would enable patients to express all the complexities of their obesity‐related complications management. This resonates with previous research in diabetes care, where it was shown that clinicians who successfully provided engagement, support, and counseling for obesity were more successful in helping patients focus on goal setting. This is dependent on the health system and the time available for multiple visits to assist with problem solving strategies, stress reduction, and psychological support.^25^

The emotional and physical consequences of obesity impacted participants' treatment preferences. The increased mass of adipose tissue and the metabolic effects of fat cells cause various issues, such as obesity stigma, sleep apnea, and osteoarthritis–which affect mobility. In addition, insulin resistance, a common consequence of obesity, is a gateway to diabetes and other complications.^26^ The participants in this study reported that as their health deteriorated, the participants were more likely to engage with options that would resolve their medical issues quickly, especially if the deterioration affected other treatments. Many patients with obesity struggle with emotional issues of low self‐esteem, quality of life, and poor body image, and this plays a role in seeking treatment.^27^ Indeed, when the topic of weight is discussed, words matter. Many patients felt judged, blamed, labeled negatively, and often self‐stigmatized.^28^

There is a need to build a greater understanding of how to leverage treatments, such as behavioral counseling and pharmacotherapy, to improve obesity‐related health outcomes.^29^ It has been shown that the knowledge of obesity is low among healthcare professionals and can be improved by educational strategies.^30^ Wynn et al. (2018) investigated the link between obesity prejudice and knowledge of obesity, among healthcare professional (HCP) groups. The researchers received 436 responses, 372 of which were complete and analyzed. These researchers found that medical specialists have the highest obesity prejudice score.^30^ Most physicians (91%) understand that 1) obesity is a disease, 2) body fat mass is at least partly regulated in the hypothalamus (70%), and 3) obesity is due to disorders of appetite regulation (69%).^31^ However, despite understanding the science around obesity, physicians indicated that obesity is due to a lack of self‐control (47%) and laziness (22%).^31^ In addition, physicians felt they did not have adequate training in obesity management.^32^ To ensure informed and collaborative decision‐making, the provision of professional development and training can be used to improve the knowledge surrounding obesity and its management.^32^

Health literacy is essential for patients who are managing chronic diseases and ensures that the patients can understand, evaluate, and use health information.^33^ Improving health literacy for patients requires that healthcare professionals ask questions to ensure adequate understanding, as well as information repeated by patients to ensure sufficient information transfer. Providing training or education for patients on their condition and the value of self‐care can also help increase confidence and support engagement.^34^ Our participants positively viewed the video played in this study, where each treatment option was explained in equipoise. Implementing such an approach as part of patient care could be beneficial, as it would provide the balanced information needed to make informed decisions about treatment.

Patient choices and decisions are influenced by their values, and these play an important role in health care decision making in collaboration with evidence‐based medicine. However, these decisions are value laden.^35^ These values are often expressed in language.^35^ The common societal response to obesity–that it is within the person's control and that the idea that social pressure can be effective toward change–is fundamentally flawed. Indeed, weight stigma has the opposite effect.^36^ When people with obesity experience weight stigma in the medical setting, these patients are more likely to cancel appointments and avoid future care.^37^ The medical community is still divided; 54% of 1096 doctors in the UK supported measures to deny treatment to patients with obesity (as well as smokers). The largest challenge is achieving medical cohesion about obesity, so that it is identified as a disease without prejudice.^38^ Social cohesion should be the foundation of national policies and political decisions that provide equal opportunity and inhibit disparity and social exclusion.^38^ Education for healthcare professionals on obesity management and the essential provision of multi‐disciplinary care for patients is a suggested approach to improve understanding and communication between the patients and health professionals.

The main guide for adequate sample size in qualitative studies is to achieve saturation of the themes which emerge.^39^ The strength of the study is the use of the participatory action method to collect data from multiple sources, facilitating validation of the results by triangulation in future projects.^11^ One of the limitations of the study was that the first author completed all the interviews and, as such, needed to be aware of their own bias when conducting research. The first author was however not involved in the informational video describing the treatment options in equipoise. To mitigate against any bias, the researcher was clear about the questions being asked, the interaction was standardized and some of the other authors assisted in the analysis of the themes.

In addition, another limitation was the risk that the participants reported what they perceived the researchers wanted to hear. This was however mitigated by the researcher conducting the interviews not being involved in the informational video and that all information was provided in equipoise, thus making it clear that there were no preferences from the research team as to the choice of options.^40^ It is important to acknowledge that as with all qualitative research, it is a challenge for this data to be extrapolated to other health systems; however, given the similar outcomes of the ACTION, ACTION‐Canada, and ACTION‐IO studies, despite very different geographical areas, it is reasonable that the themes we elucidated will be replicated in other healthcare systems.

## 5 CONCLUSION

Patient preferences play an essential role in the adherence to treatment and improved treatment outcomes. Providing a structure that allows an informed decision making between both the patient and healthcare professional may help to establish the optimal approach for patients. Factors that influence decisions on obesity treatment were 1) structural factors of the healthcare setting, such as access and cost, 2) autonomy, including lack of knowledge and the perception of not being heard, 3) interaction with formal care, experienced as a lack of knowledge and support, and 4) emotional and physical consequences of obesity, including fear of side effects of treatment. These challenges can be addressed by increasing knowledge and improving support by healthcare professionals, improving the health literacy of patients, and improving access to affordable treatment.

Given these findings, it is hoped that future obesity care will be more patient‐centric and involve a more informed decision making along the healthcare pathway to improve adherence and patient outcomes. Future solutions may include 1) increased health education for healthcare professionals in primary care and hospital settings, 2) creating educational programmes and information platforms for patients to improve health literacy about obesity and its complications, and 3) expansion of obesity treatment options–with proven health economic benefits–to ensure equitable, society‐wide access to disease management and support. Future research on health policy should focus on improving access to treatment pathways for patients as well as developing health literacy programs and educational programs for healthcare professionals.

## AUTHOR CONTRIBUTION

Hilary Craig: Conceptualization, formal analysis, funding acquisition, methodology, Literature search, figures, study design, data collection, data analysis, data interpretation, writing, review and editing. Prof Carel le Roux: Conceptualization, formal analysis, funding acquisition, methodology, supervision, validation, writing review and editing. Dr Dalal Alsaeed: Conceptualization, formal analysis, funding acquisition, methodology, supervision, validation, writing review and editing. Suzanne Norris: Formal analysis, review and editing. John Holian: Formal analysis, review and editing. Cormac Kennedy: Formal analysis, review and editing. Alix Feldman: Formal analysis, review and editing. SOPHIA (the Stratification of Obesity Phenotypes to Optimize Future Obesity Therapy (SOPHIA) project (www.imisophia.eu)) Review Committee: Formal analysis, review and editing.

## CONFLICT OF INTEREST STATEMENT

Hilary C. Craig, Part funding of PhD tuition fees from the Stratification of Obesity Phenotypes to Optimize Future Obesity Therapy (SOPHIA) project (www.imisophia.eu). Dalal Alsaeed—no conflict of interest. Suzanne Norris—no conflict of interest. John Holian—no conflict of interest. Cormac Kennedy—no conflict of interest. Alix Feldman—employed by Novo Nordisk. Carel W. le Roux: Consulting fees/Honoria/Support for meetings: NovoNordisk, Eli Lilly, Johnson & Johnson, Boehringer Ingelheim, GI Dynamics, Herbalife. Leadership/fiduciary role in Board: Irish Society for Nutrition and Metabolism (unpaid). Stock Options: Keyron Previous Chief medical officer and Director of the Medical Device Division of Keyron in 2011. Both of these were unremunerated positions. Previous investor in Keyron, which develops endoscopically implantable medical devices intended to mimic the surgical procedures of sleeve gastrectomy and gastric bypass. He continues to provide scientific advice to Keyron for no remuneration.

## Supporting information

Supporting Information S1
