# Supplementary material for: Patient perspectives about treatment preferences for obesity with complications
Source: Obes Sci Pract. 2023 Oct 29;10(1):e720. doi: 10.1002/osp4.720 (PMC10804341; doi:10.1002/osp4.720)
Supplement: Supplementary file 1 — Supporting Information S1 [file OSP4-10-e720-s001.docx]

**Appendix 1: Themes, Sub-Themes and Participants’ Quotes**

| **Themes** | **Sub-Themes** | **Condition** | **Participants’ Quotes** |
| --- | --- | --- | --- |
| Structural Factors Influencing Choice | Access | T2D | *‘But the access to a dietitian, the access to a nutritionist is not necessarily very evident or there. that holistic piece from maybe a dietitian or a nutritionist talking to you as part of the treatment program for a chronic illness.’* |
|  | Cost | T2D | *‘Now I'm on two needles. I've got the second. It’s costing me €180 a month for the second needle. Now, I find that fairly hard. I’m not working, and I haven’t worked for years, because of my issues that I have.’* |
| Autonomy | Knowledge and Information | NAFLD | *‘I suppose I’d be in a lack of knowledge about it. that I wouldn’t know enough and maybe, it’s a big jump. It’s a big leap into the unknown. And obviously I’d like more knowledge and maybe more research into all of these things.’* |
|  | Not Being Heard | CKD | *‘Yeah, exactly. And it's like you're looked at as if it's all completely your fault. I know some of it is, you know what I mean? It could be 70% your own fault. But when you're asking for help it's kind of like, "Just go away and do that, and then come back to me." It's always it's like that's the answer. "When you do that, come back to me then.’* |
|  | Support | NAFLD | *‘That’s the hardest part I would find. I would love the help of and guidance and medical supervision of someone who really knows what they’re talking about and can maybe educate me perhaps people like me, you know. There’s nothing going to…, for me anyway.’* |
| Interaction with Formal Care | Knowledge and Information | NALFD/T2D | *‘I was just told, you know go to Weight Watchers, you know, start losing weight. I was never given, I mean, was told to google it, have a look online. It is very difficult to do that when you’ve got, when you don’t really understand what you are reading.* |
|  | Support | NAFLD | *‘You know, such a waste of time, you know, and you think you go to someone who's going to help you, and they are just “yeah” looking at the clock and you know just took it and you know why? Because they are overburdened with it because there's so many people looking for psychological help that they cannot save, or they cannot help everyone, and it costs at the moment.’* |
| Emotional and Physical Consequences of Obesity | Emotional Impact | CKD | *‘You know, as I said to you that, like I do suffer from low confidence. You know so I keep my head down I don't really, I try and stay out of the crowd you know because I don't want the attention on me, you know.’* |
|  | Physical Impact | T2D | *‘I was diagnosed with diabetes in about 2006 or 2005 and then I have tried to diet. I’ve been to one or two dietitians and that kind of thing. I’ve told them about my diet, and I’ve tried to cut down and cut back and that kind of thing. But the only thing is, I have arthritis in both knees from wear and tear and I can’t walk.’* |
|  | Support – Family and Friends | NAFLD | *‘People would say look don’t be worrying about that, don’t be worrying about that sure aren’t you fit you’re healthy you’re alive for years and I remember going in for Chemo and like one of the first things my mother asked them was before I went in for treatment is does she need to lose weight you know like do I need to lose weight before I go in for chemotherapy because that’s what I’ve lived with all my life’* |
|  | Side-effects | CKD | *‘I would've taken the view that the value of the medication was less, relative to the side effects than working on a diet basis. And so, if those risks were reduced and those side effects were less, then yeah.’* |
